# One-step *in situ* growth of ZnS nanoparticles on reduced graphene oxides and their improved lithium storage performance using sodium carboxymethyl cellulose binder†

**DOI:** 10.1039/c8ra00470f

**Published:** 2018-03-01

**Authors:** Lun Lu, Liwei Jing, Zhizheng Yang, Guangyu Yang, Cheng Wang, Jinguo Wang, Huiyuan Wang, Qichuan Jiang

**Affiliations:** Key Laboratory of Automobile Materials of Ministry of Education, Department of Materials Science and Engineering, Jilin University No. 5988 Renmin Street Changchun 130025 PR China wanghuiyuan@jlu.edu.cn jgwang@jlu.edu.cn +86 431 8509 4699; Institute of Scientific and Technical Information of Jilin Province No. 940, Shenzhen Road Changchun 130033 PR China; State Key Laboratory of Automotive Simulation and Control, International Center of Future Science, Jilin University Changchun 130012 PR China

## Abstract

ZnS nanoparticles are *in situ* grown on reduced graphene oxides (rGO) *via* a simplified one-step hydrothermal method. Sodium carboxymethyl cellulose (CMC) is firstly applied as the binder for ZnS based anodes and shows a more advantageous binding effect than PVDF. To simplify the synthesis procedure, l-cysteine is added as the sulfur source for ZnS and simultaneously as the reducing agent for rGO. The average diameter of ZnS nanoparticles is measured to be 13.4 nm, and they uniformly disperse on the rGO sheets without any obvious aggregation. As anode materials, the CMC bound ZnS–rGO nanocomposites can maintain a high discharge capacity of 705 mA h g^−1^ at a current density of 500 mA g^−1^ for 150 cycles. The significantly improved electrochemical performance mainly derives from the combined effects of the small and uniformly dispersed ZnS nanoparticles, the high conductivity and structural flexibility of rGO and the strong binding ability of CMC.

## Abstract 2

ZnS nanoparticles are *in situ* grown on reduced graphene oxides (rGO) *via* a simplified one-step hydrothermal method.

## Graphical abstract

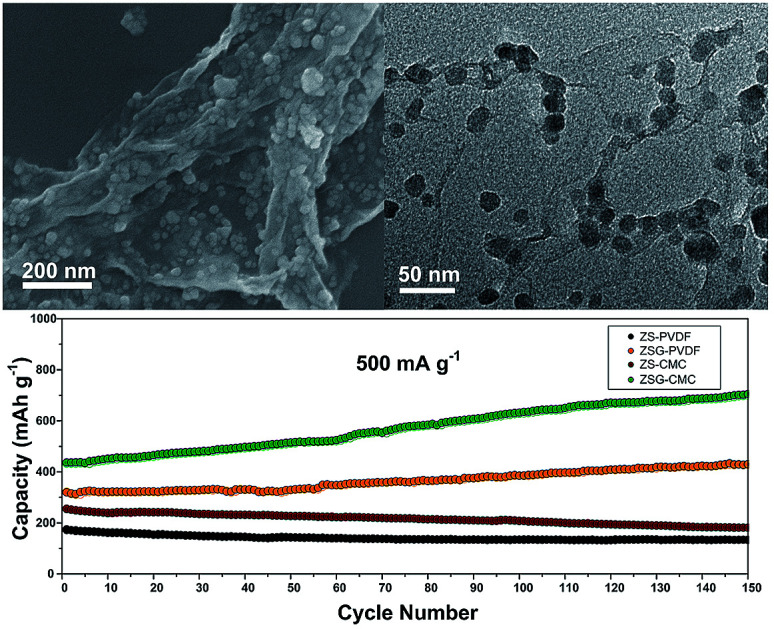

## Introduction

The ever growing demand for green and high-efficiency energy storage devices has greatly promoted the development of the lithium-ion batteries (LIBs) industry. However, the power and energy density of current commercial LIBs still cannot satisfy the high requirements of next generation batteries which are expected to be applied in electric vehicles.^1,2^ Novel electrode materials, especially high-performance anodes, are urgently needed to increase the overall electrochemical performance of LIBs. Among the vastly investigated anode materials, ZnS is a promising candidate as it possesses a high theoretical capacity of 962 mA h g^−1^.^3^ The naturally abundant zinc and sulfur are cheap and environmental friendly, which makes ZnS anodes even more advantageous. But the practical application of ZnS based anode materials is still hindered by their low rate capability and severe capacity decay which are caused by their low conductivity and huge volume expansion during cycling. Thus it is imperative to find effective methods to solve these problems.

For ZnS anode materials, reducing the particle size to the nanometer scale and compositing ZnS with carbon materials are two adoptable strategies to improve their long-term cycling stability and rate performance. Nanosized materials, especially nanoparticles and nanodots, are quite advantageous as their shortened ion and electron transport path will greatly facilitate the lithium storage process at high current rate.^4–6^ And among the various carbon based materials, reduced graphene oxide (rGO) is an ideal performance-enhancing additive because of its high electrical conductivity, large surface area and excellent structural flexibility.^7–9^ Actually, the advantages of the above two strategies can be combined through the fabrication of composite materials which contain rGO nanosheets and uniformly dispersed ZnS nanoparticles. But synthesizing such ZnS–rGO nanocomposites with desired particle size and morphology is not an easy task because zero-dimensional nanoparticles often suffer from severe aggregation problems. Aggregated particles will block the penetration of electrolyte, decrease the ion transport efficiency and lower the utilization of active materials.^10–12^ Moreover, rGO is usually obtained by the reduction of graphene oxide (GO), which means the need for extra reducing agent (like toxic hydrazine) or additional thermal annealing process.^13,14^ And how to guarantee the firm adhesion of ZnS nanoparticles on the rGO sheets is another problem that should be considered. Physically adsorbed particles can be easily peeled off from rGO, which will result in the loss of cell capacity. Considering these issues, it is obvious that green and simplified method is still urgently needed to fabricate stable ZnS–rGO nanocomposites while ensuring the uniform dispersion and firm adhesion of ZnS particles.

In addition to optimizing ZnS anodes from the structural and materials preparation aspects, choosing suitable binder for ZnS is also of great importance. Traditional PVDF binder has been proved to be inappropriate for anode materials based on alloying or conversion mechanism because it is difficult for PVDF to withstand the huge volume expansion that is involved.^15,16^ So PVDF also seems not to be a good choice for ZnS since alloying and conversion reactions are both existed in its charge–discharge process. Compared with PVDF, sodium carboxymethyl cellulose (CMC) is probably more suitable for ZnS as it can offer a homogeneous 3D networking around the active materials, endowing the electrode with stronger ability to accommodate mechanical stress.^17^ The successful application of CMC in Si and metal oxides anodes further demonstrates its advantage.^18–20^ However, to the best of our knowledge, whether CMC binder has a positive effect on ZnS anodes has not yet been reported. Figuring this out would be meaningful as it may provide an easy and scalable way to improve the cycling performance of ZnS.

Based on the above considerations, herein we develop a simplified one-step hydrothermal route to *in situ* grow ZnS nanoparticles on rGO and firstly use CMC as the binder for ZnS based anodes. In the synthesis procedure, l-cysteine is added as both the sulfur source for ZnS and simultaneously as the reducing agent for rGO. No extra annealing treatment or reagent is needed to complete the reduction process. The introduced rGO has proved itself to be a desirable matrix as it can improve the dispersity of ZnS and enhance the conductivity and structural stability of the as-prepared composite materials. The binding effect of CMC has been compared with traditional PVDF binder. With the synergistic effects of the flexible rGO nanosheets, firmly attached ZnS nanoparticles and the advantageous CMC binder, the ZnS–rGO nanocomposites exhibit a much enhanced electrochemical performance.

## Experimental

### Material preparation

Analytical-grade Zn(CH_3_COO)_2_·2H_2_O, l-cysteine were used as received without further purification. GO was prepared by the oxidation of natural flake graphite powder by a modified Hummers method.^21^ Typically, 3 mmol Zn(CH_3_COO)_2_·2H_2_O was dissolved into 50 ml deionized water under magnetic stirring for 10 minutes. Then 10 ml of 2.0 mg ml^−1^ GO aqueous suspension was added and treated by ultrasonication for 30 minutes. Subsequently, 4.5 mmol l-cysteine was dissolved into the above solution. After being vigorously stirred for 20 minutes, the resulting mixture was transferred into a Teflon-lined stainless steel autoclave and heated to 180 °C for 12 h. The product was collected by centrifugation and washed thoroughly by deionized water and absolute ethanol. After being dried in air at 60 °C for 12 h, the ZnS–rGO nanocomposites (denoted as ZSG) were obtained. As control samples, pure ZnS (denoted as ZS) and rGO were also prepared using the same method without the addition of GO suspension or Zn(CH_3_COO)_2_·2H_2_O.

### Material characterization

The crystallographic structure and phase purity of as-prepared samples were characterized by X-ray diffraction (XRD, *D*_max_/2500 PC, Rigaku, Japan) with Cu Kα radiation (*λ* = 1.5406 Å). ESCALAB 250 spectrometer with Al Kα X-ray source was used to collect the X-ray photoelectron spectroscopy (XPS) data. The morphology of the as-prepared samples is observed by field emission scanning electron microscopy (FESEM, JSM-6700F, Japan). Thermogravimetric analysis (TG, SDT-Q600, TA instruments Inc. USA) was carried out under air flow (100 ml min^−1^) at a heating rate of 10 °C min^−1^. Raman spectra measurements were recorded on a Renishaw InVia Raman microscope with an excitation wavelength of 633 nm. TEM and HRTEM images were obtained by a FEI-TECNAI G2 F20/America microscope.

### Electrochemical measurements

The working electrode is prepared by mixing active materials, acetylene black and polyvinylidene fluoride (PVDF) or sodium carboxymethyl cellulose (CMC) to form a homogenous slurry. The weight ratio of the active materials, conducting agent and binder is 7 : 2 : 1. The slurry was uniformly pasted on a copper foil and then dried at 70 °C for 12 h in a vacuum oven. The electrode materials prepared with PVDF is denoted as ZS–PVDF, ZSG–PVDF and rGO–PVDF. Similarly, electrode using CMC as the binder is denoted as ZS–CMC, ZSG–CMC and rGO–CMC. CR2025-type half-coin cells were assembled in an argon filled glove box with H_2_O and O_2_ contents below 1 ppm. Li foil is used as the counter and reference electrode. Ethylene carbonate (EC), ethyl methyl carbonate (EMC) and dimethyl carbonate (DMC) were mixed in a volume ratio of 1 : 1 : 1, and 1 M LiPF6 was dissolved in the mixture, which was used as the electrolyte. A LAND CT2001A battery instrument was applied to perform galvanostatic charge–discharge tests at various current densities in 0.01–3.0 V. The cyclic voltammetry (CV) tests were conducted in 0.01–3.0 V at a scan rate of 0.1 mV s^−1^ on a CHI650D electrochemical workstation. Electrochemical impedance spectra (EIS) measurements were also recorded on the CHI650D electrochemical workstation with an AC signal amplitude of 5 mV from 0.01 Hz to 100 kHz.

## Results and discussion

### Structure and morphology

**Fig. 1:**
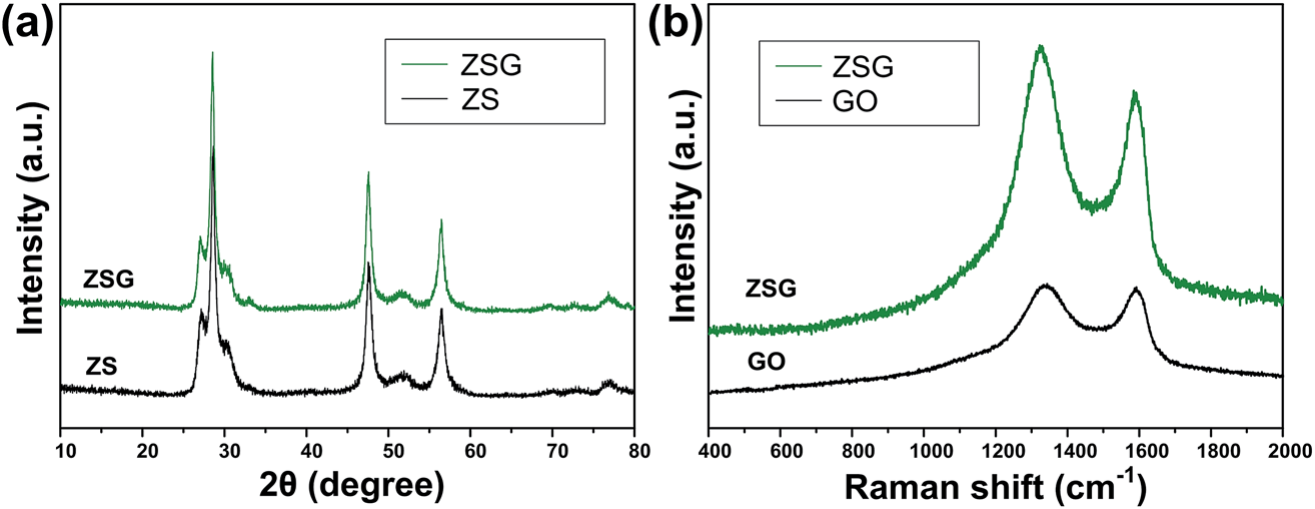
(a) XRD patterns of ZS and ZSG. (b) Raman spectra of ZSG and GO.

The crystal structure and phase purity of the as-prepared products were characterized by XRD (Fig. 1a). All the peaks of the two samples correspond well with wurtzite structured ZnS (JCPDS card no. 39-1363). No obvious difference is observed in the XRD patterns of pure ZnS sample (denoted as ZS) and ZnS–rGO sample (denoted as ZSG). The peak related with rGO is not observed in ZSG, which may result from its relatively low content in the composites. No additional diffraction peaks from impurities are found, indicating the high purity of the samples. To confirm the successful reduction of GO by l-cysteine, Raman spectroscopy measurements were carried out on ZSG and pristine GO powder. The Raman spectra displays disorder-induced D band (∼1350 cm^−1^) and tangential G band (∼1590 cm^−1^) in Fig. 1b. The *I*_D_/*I*_G_ value of ZnS–rGO is 1.14, and the value for GO is 1.03. The increased *I*_D_/*I*_G_ value indicates the successful reduction of GO as it can decrease the average size of the sp^2^ domains.^22,23^ And among all the reagents, only l-cysteine possesses the ability to reduce GO.^24^ Thus it is obvious that l-cysteine, with its strong reducibility and excessive amount, has successfully reduced GO to rGO.

**Fig. 2:**
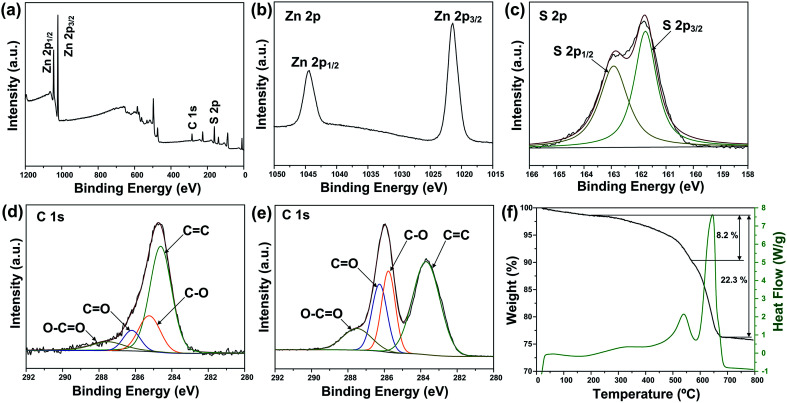
(a) XPS survey spectra and high-resolution XPS spectra of (b) Zn 2p, (c) S 2p and (d) C 1 s of ZSG. (e) High-resolution XPS spectra of C 1 s of GO. (f) TGA and DSC curves of ZSG.

X-ray photoelectron spectroscopy (XPS) analysis was carried out to further verify the reduction of GO and investigate the oxidation state of the component elements in ZSG. As shown in Fig. 2a, Zn, S and C elements are detected in the widescan spectrum. Typical Zn 2p_1/2_ and Zn 2p_3/2_ peaks are found at 1044.5 eV and 1021.4 eV in the high-resolution spectra of Zn 2p in Fig. 2b.^25^ For the S 2p spectra in Fig. 2c, the two peaks located at 163.1 eV and 161.8 eV can be attributed to the S 2p_1/2_ and S 2p_3/2_ spin orbit peaks of ZnS. For ZSG, the successful reduction of GO can also be proved by the change of C 1 s XPS spectra. Four peaks are detected in the C 1 s spectra of unreduced GO in Fig. 2e. The peak centered at 283.7 eV can be assigned to the C=C bond. The binding energy of the C=C bond is slightly deviated from the usually reported value (∼284.6 eV).^26^ This might be caused by the modification of the electronic structure of GO by some electronic acceptor molecules. As reported by Mullen, the electronic donor and acceptor molecules within the functional groups on graphene can modify its electronic structure, which can lead to the upshift/downshift of the carbon peaks in the XPS spectra.^27^ Moreover, the defective carbon structures on GO also possess the ability to reduce the binding energy of C=C bond and cause the downshift of the XPS peak.^28^ Considering the complex electronic and defective structures of GO, further investigation is still needed to figure out the detailed mechanism. The other three peaks at 285.8, 286.3 and 287.5 eV are typical C–O, C=O and O–C=O bonds in GO.^29^ In contrast, the peak intensity of these oxygen-containing bonds decreases dramatically in the C 1 s spectra of ZSG (Fig. 2d), indicating that most of them are removed through the reduction process. Compared with GO, rGO usually possess better conductivity, which is favorable in improving the performance of the electrode materials.^30^ Thermogravimetric analysis (TGA) and differential scanning calorimetry (DSC) were conducted in air to evaluate the rGO content in the composites (Fig. 2f and S1†). The mass loss before 200 °C can be ascribed to the evaporation of adsorbed water. For the ZS sample (Fig. S1†), the oxidation of ZnS has led to a total mass loss of about 17.5%, quite close to the theoretical value. For ZSG, the weight loss between 200 and 568 °C is associated with the combustion of rGO and the weight loss after 568 °C corresponds to the oxidation of ZnS. From the TGA results, the rGO content in the ZSG nanocomposites is measured to be 8.2%.

**Fig. 3:**
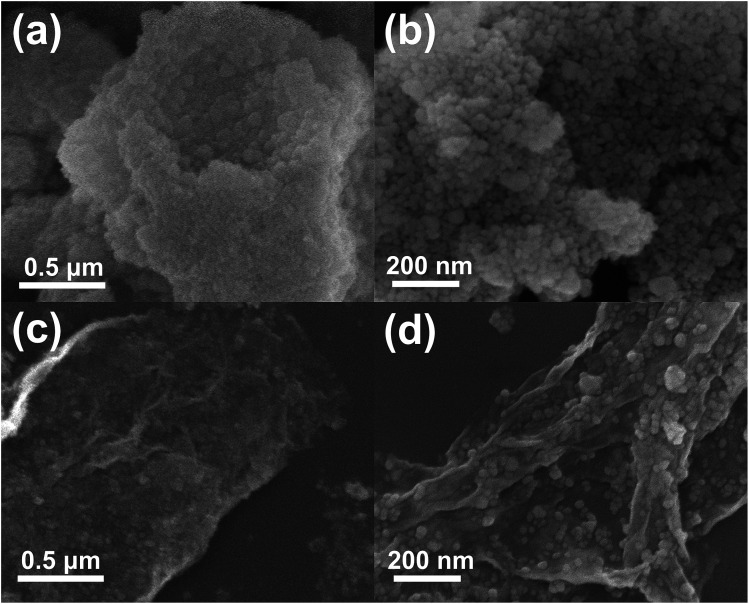
Low and high magnification FESEM images of ((a) and (b)) ZS and ((c) and (d)) ZSG.

**Fig. 4:**
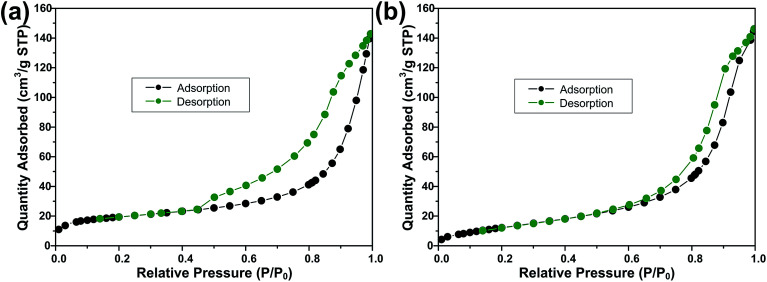
N_2_ adsorption–desorption isotherms of (a) ZSG and (b) ZS.

Fig. 3 shows the FESEM images of ZS and ZSG. Without rGO, pure ZnS nanoparticles suffer from severe aggregation. A lot of large agglomerates are observed in Fig. 3a and b. Aggregated nanoparticles will impede the infiltration of electrolyte, thus lowering the utilization of active materials during cycling. In stark contrast, ZnS nanoparticles in ZSG are uniformly and firmly anchored on rGO nanosheets and almost no aggregation is observed (Fig. 3c). Such morphology is quite beneficial for the penetration of electrolyte. As shown in Fig. 4, the specific surface area of the ZS and ZSG samples were characterized by N_2_ adsorption–desorption measurement. Type IV isotherm with a distinct hysteresis loop in relative pressure of 0.45 < *P*/*P*_0_ < 1.00 is observed in Fig. 4a and b. The BET specific surface area of the ZS and ZSG are 42.5 and 65.4 m^2^ g^−1^, respectively, proving that the introduction of rGO has increased the surface area of the composites. Large surface area is beneficial as it can increase the contact area between the electrode and electrolyte and provide more active sites for electrochemical reactions.^31^ It is apparent that the ZSG sample, with the *in situ* formed ZnS nanoparticles and the wrinkled rGO nanosheets, possesses a more advantageous nanostructure than the ZS sample.

**Fig. 5:**
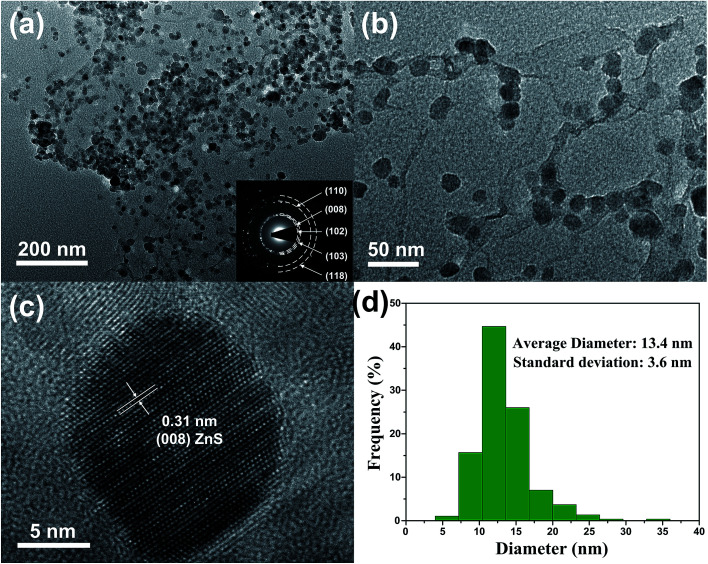
(a) Low and (b) high magnification TEM images, (c) HRTEM image and (d) particle size distribution of ZSG. Inset of (a) is the corresponding SAED pattern.

The TEM images in Fig. 5 can provide more detailed morphological information of ZSG. In Fig. 5a, numerous ZnS nanoparticles are homogeneously distributed on a single rGO nanosheet, agreeing well with the nanostructures observed in the FESEM images. Interestingly, part of the particles are wrapped by the rGO and each particle can be clearly discerned in Fig. 5b. For these nanoparticles, the stress from volume expansion will be easily accommodated because of the high elasticity of the rGO layer.^32^Fig. 5c shows the HRTEM image of a single ZnS nanoparticle. The interplanar distance of the lattice fringes is 0.31 nm, corresponding to the (008) plane of ZnS. The polycrystalline nature of ZnS is confirmed by the SAED pattern in the inset of Fig. 5a. The diffraction rings can be assigned to the (110), (008), (102), (103), (118) planes of ZnS. Fig. 5d displays the particle size distribution of ZnS in the composites, which is obtained from the Nano Measurer software by counting 300 ZnS particles in Fig. 5a. The size of most ZnS particles is in the range of 7–20 nm, and the average diameter is calculated to be 13.4 nm with a standard deviation of 3.6 nm. Such small particle size, combined with the excellent electronic conductivity of rGO, will significantly enhance the ion and electron transport efficiency, thus improving the rate capability of the ZSG sample.

### Electrochemical performance

**Fig. 6:**
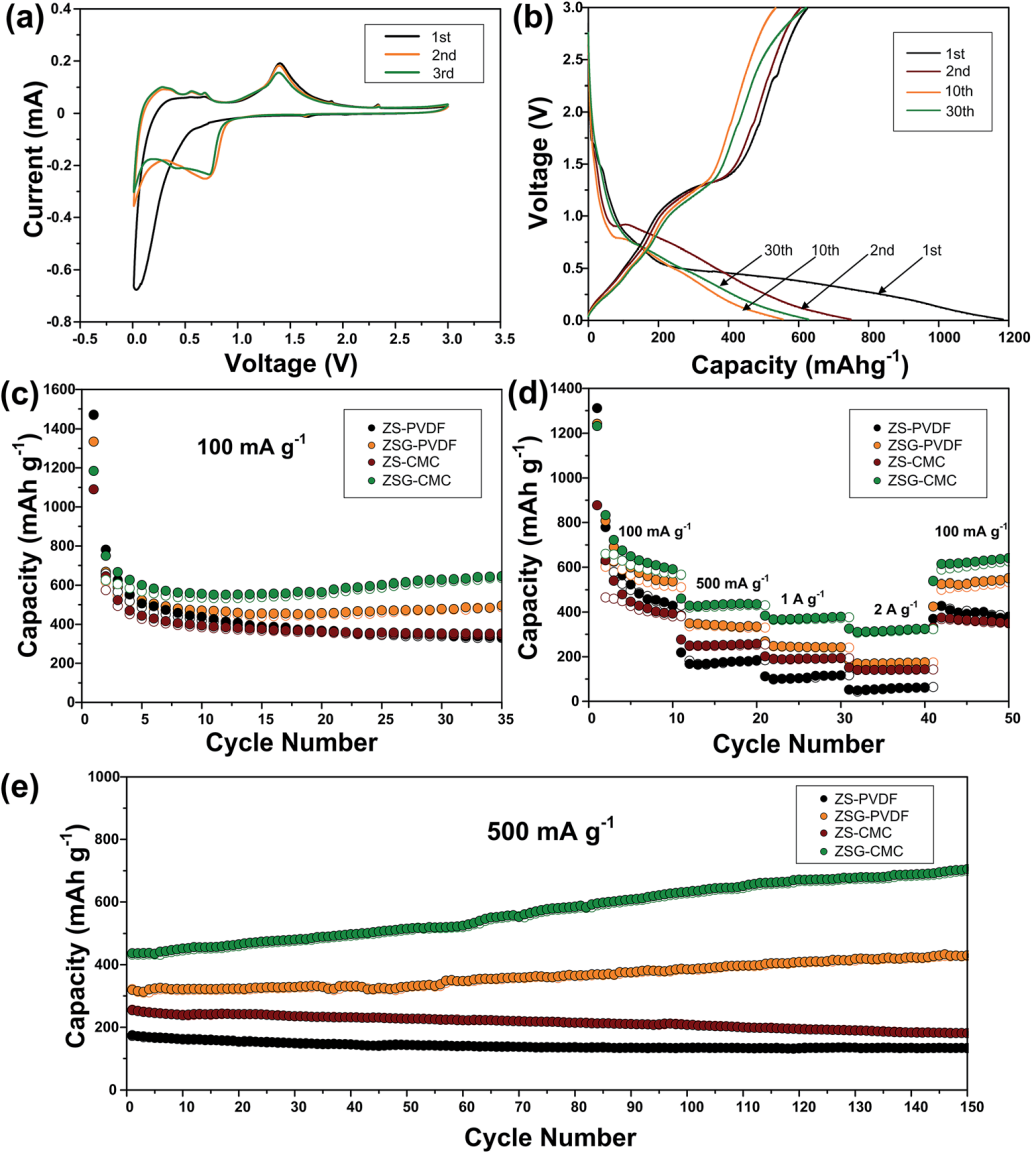
Electrochemical performance of ZS–PVDF, ZSG–PVDF, ZS–CMC and ZSG–CMC. (a) Cyclic voltammetry curves of ZSG–CMC in the voltage range of 0.01–3 V at a scan rate of 0.1 mV s^−1^. (b) Galvanostatic charge–discharge profiles of ZSG–CMC at 100 mA g^−1^. (c) Cycling performance and (d) rate capability of ZS–PVDF, ZSG–PVDF, ZS–CMC and ZSG–CMC. (e) Continuous long-term cycling performance of the above four samples after the rate test.

To investigate the electrochemical performance and compare the binding effect of CMC and PVDF, ZS and ZSG electrode materials are prepared with both the two sorts of binders, and they are denoted as ZS–PVDF, ZSG–PVDF, ZS–CMC and ZSG–CMC, respectively. As shown in Fig. 6a, the cyclic voltammogram (CV) test of ZSG–CMC was carried out at a scan rate of 0.1 mV s^−1^ in 0.01–3 V. During the first cathodic scan, the broad and weak reduction peak below 0.8 V is related with the reduction of ZnS to metallic zinc and the formation of Li_2_S.^33^ Besides, the Li–Zn alloying process and formation of SEI film also take place in this voltage range.^34^ During the first anodic scan, three small peaks are observed in 0.01–0.7 V, which can be assigned to the multi-step dealloying process of Li–Zn alloys. The oxidation peak at ∼1.4 V corresponds to the back conversion of metallic zinc to ZnS. The corresponding reversible reactions are summarized as follows:

1 ZnS + Li^+^ + 2e^+^ ↔ Zn + Li_2_S

2 Zn + *x*Li^+^ + *x*e^+^ ↔ Li_*x*_Zn

Interestingly, in the first scanning cycle, one tiny cathodic peak at ∼1.6 V and two anodic peaks at ∼1.8 V and ∼2.3 V are observed and they gradually disappear in subsequent cycles. This can be ascribed to the insertion and extraction of a small amount of Li^+^ into the nanocomposites. Such phenomenon is also reported in other metal oxides with small particle size.^35–37^ The irreversible formation of SEI film has led to the change in the curve shape of the second cathodic scan. The peaks corresponding to the reduction of ZnS has right shifted to voltage above 0.8 V. The CV curves coincide well from the second cycle onwards, indicating the good reversibility of ZSG. As for the CV peaks of rGO, the insertion of Li^+^ should appear around 0.5 V and the extraction of Li^+^ from rGO happens in a wide voltage range from 0.05 to 3 V.^13^ However, the overlap of the reaction voltage with ZnS and the low content of rGO in the nanocomposites has made the peaks corresponding to rGO invisible in Fig. 6a. Fig. 6b shows the galvanostatic charge–discharge profiles of ZSG–CMC at 100 mA g^−1^ in 0.01–3 V. The slope ranging from 0.01–0.7 V in the first discharge curve and the plateau around 1.4 V in the first charge curve are consistent with the above CV results. During the first cycle, the ZnS–rGO nanocomposites deliver an initial discharge and charge capacity of 1184 mA h g^−1^ and 625 mA h g^−1^, exhibiting a relatively low coulombic efficiency of 55.1%. The large capacity loss is mainly due to the formation of the solid electrolyte interphase (SEI) layer.^38^ In the 2nd, 10th and 30th cycle, the coulombic efficiency has increased to 80.9%, 96.2% and 98.7%, demonstrating the high reversibility of the electrode materials.

The cycling performance of ZS–PVDF, ZSG–PVDF, ZS–CMC and ZSG–CMC at 100 mA g^−1^ is displayed in Fig. 6c. With PVDF as binder, ZS and ZSG show an initial discharge capacity of 1471 mA h g^−1^ and 1334 mA h g^−1^. Then the capacity of ZS–PVDF gradually decreases and drops to 337 mA h g^−1^ in the 35th cycle while the ZSG–PVDF sample can maintain a stable capacity of 496 mA h g^−1^. This suggests that the incorporation of rGO with ZnS has effectively helped to buffer the volume expansion, making the electrode more stable during charge–discharge cycles. When CMC is used as the binder, the cycling performance of ZSG is further improved. It can deliver an initial discharge capacity of 1184 mA h g^−1^ and maintain at 645 mA h g^−1^ after 35 cycles, retaining 54.5% of its initial capacity, which is much higher than that of ZSG–PVDF (37.2%). The improved capacity retention may mainly derive from the stronger binding ability of CMC than PVDF.^18^ During cycling, the carboxylic groups in CMC can form hydrogen binding with ZnS nanoparticles, alleviating pulverization and therefore increase the utilization rate of the active materials. Though the content of rGO in the nanocomposites is low and its contribution to the capacity of the electrode is limited, investigating the performance of pure rGO anodes is necessary and would be conducive to the understanding of the electrochemical properties of ZSG. The cycling performance of rGO–PVDF and rGO–CMC is shown in Fig. S2a.† The rGO–CMC sample exhibits an initial discharge capacity of 1147 mA h g^−1^ and maintained at 574 mA h g^−1^ after 35 cycles at 100 mA g^−1^, while the rGO–PVDF can also retain a stable capacity of 443 mA h g^−1^.

The advantage of CMC binder becomes more obvious when the samples are tested at high current rate. Fig. 6d shows the rate performance of the four samples tested at various current densities ranging from 100 mA g^−1^ to 2 A g^−1^. ZS–PVDF can only retain 52 mA h g^−1^ and ZSG–PVDF can hold a capacity of 174 mA h g^−1^ at 2 A g^−1^. The improved rate performance of ZSG–PVDF mainly derives from the good conductivity of rGO and the uniformly dispersed nanosized ZnS particles. On the one hand, the problem of low conductivity of ZnS will be ameliorated after the introduction of rGO. On the other hand, the small particle size and uniform dispersion of ZnS will shorten the diffusion length of ions and electrons, offer larger surface area for reactions and facilitate the penetration of electrolyte. Interestingly, ZS–CMC shows a much inferior capacity than ZSG–PVDF at low current density, but the capacity gap narrows with the increase of current and even becomes comparable at 2 A g^−1^. The low-density PVDF binder possess a porous structure. Such structure can guarantee a good immersion of active materials in the electrolyte. That is why the PVDF bound ZSG shows comparable electrochemical performance at low current density. However, according to Winter *et al.*, the porous nature of PVDF will result in a low electronic conductivity, thus weakening the electrode performance under high current rate.^39^ In comparison, the relatively compact CMC binder is not faced with this problem. Its performance under high rates is only limited by the Li diffusion coefficient within the electrode materials. Moreover, there are also reports showing that CMC could modify the Li diffusion coefficient and accelerate the ion transport, thus leading to a more advantageous rate capability.^40^ ZSG–CMC still displays the highest rate performance with capacities of 379 mA h g^−1^ at 1 A g^−1^ and 325 mA h g^−1^ at 2 A g^−1^. Moreover, its capacity can recover to 640 mA h g^−1^ after the current is changed back to 100 mA g^−1^. For pure rGO (Fig. S2b†), the rate performance is not satisfactory as the rGO–CMC can only maintain a capacity of 145 mA h g^−1^ at 2 A g^−1^, much inferior than the ZSG sample. The high performance of ZSG and the poor rate capability of ZS and rGO fully demonstrate that the incorporation of ZnS and rGO has endowed the composites with extra electrochemical advantages that are not possessed by the single component.

Followed by the rate test, the samples are further cycled for another 150 cycles at 500 mA g^−1^. As shown in Fig. 6e, both ZS–PVDF and ZS–CMC suffer from a slow capacity decay and only 133 mA h g^−1^ and 181 mA h g^−1^ are retained after 150 cycles. Different from ZS samples, a gradual capacity increase phenomenon is observed for both ZSG samples. In the 150th cycle, the capacity of ZSG–PVDF has increased to 429 mA h g^−1^ while a high capacity of 705 mA h g^−1^ is retained for the ZSG–CMC sample. Such capacity increase phenomenon is commonly reported in nanosized metal oxides and sulfides.^41–43^ It is mainly caused by the reversible formation of polymeric gel-like films which can offer extra capacity through the kinetically activated electrolyte degradation.^44^ Pseudo-capacitive behaviour at the interface of metal and Li_2_O or Li_2_S nanocrystals is also believed to play an important role in increasing the capacity.^45,46^ It seems that the special conversion reaction mechanism of metal oxides/sulfides and the nano-dimensional morphology are the essential conditions to trigger the capacity increase phenomenon. This also explains why the capacity increase effect is not observed in the ZS and rGO samples. As for rGO, the long-term cycling performance of rGO–PVDF and rGO–CMC are comparable (Fig. S2c†). Both of them stabilize around 300 mA h g^−1^ after being tested for another 150 cycles. Actually, the advantage of CMC binder is not so obvious in pure rGO sample, which may be due to the absence of conversion reactions and the relatively small volume expansion during the charge–discharge process of rGO.

**Fig. 7:**
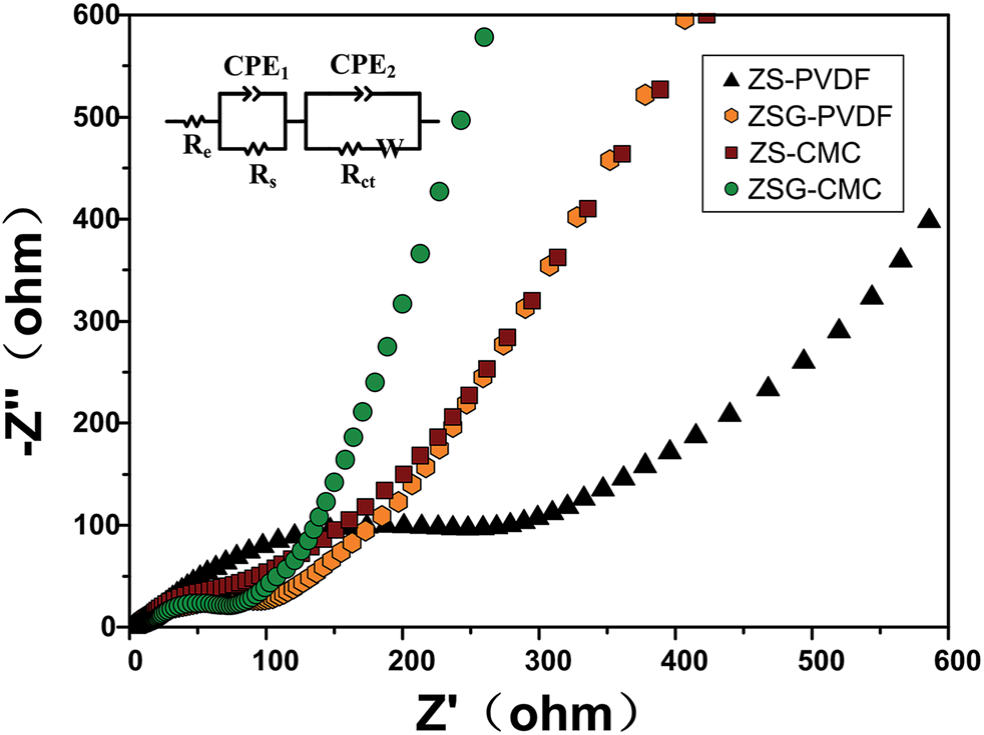
Electrochemical impedance spectra and corresponding equivalent electrical circuit (inset) of ZS–PVDF, ZSG–PVDF, ZS–CMC and ZSG–CMC.

To gain more information about the nanocomposites, electrochemical impedance spectroscopy (EIS) measurements were performed after the cells were tested at 500 mA g^−1^ for 100 cycles. The Nyquist plots of the four samples are depicted in Fig. 7. For each sample, the Nyquist plots contain two semicircles and a slope line. The high frequency semicircle is related with the resistance *R*_s_ and constant phase element CPE_1_ of the SEI film. The semicircle in the medium frequency region corresponds to the charge transfer resistance *R*_ct_ and CPE_2_ of the electrode/electrolyte interface. The Warburg impedance (*Z*_w_) which reflects the diffusion of lithium ions in electrode appears as the slope long line in the low frequency region. It can be clearly seen that the diameter of the curve of ZSG–CMC is smaller than the other three samples, indicating that the nanocomposites possess a lower charge transfer resistance and higher conductivity. This is further verified by the fitting results. The inset of Fig. 7 is the corresponding equivalent electrical circuit. According to the results, the *R*_ct_ value for ZS–PVDF, ZSG–PVDF, ZS–CMC and ZSG–CMC are 388.7 Ω, 59.9 Ω, 61.7 Ω and 47.1 Ω, respectively. Lower charge transfer resistance means a higher transport efficiency of Li^+^ ions through the interface of the electrode and electrolyte, which is conducive to the enhancement of rate capability of the electrode materials.^47^

The excellent electrochemical performance of ZSG–CMC can be ascribed to the following reasons. Firstly, the high structural flexibility and conductivity of rGO can help to alleviate the huge volume expansion and increase ion transport efficiency of the composite materials. Secondly, the small particle size and uniform dispersion of ZnS can offer larger surface area for reactions and facilitate the penetration of electrolyte, guaranteeing a high utilization of active materials. Thirdly, the conversion reaction mechanism and small size of ZnS can trigger a capacity increase phenomenon through the formation of gel-like films and pseudo-capacitive behavior. Finally, the CMC binder can provide a strong binding effect to alleviate pulverization and to some extent modify the Li diffusion coefficient to further improve the rate capability. These advantages have combined to enhance the overall cycling stability and rate capability of the ZSG–CMC electrode.

## Conclusions

In summary, ZnS–rGO nanocomposites are synthesized *via* a simplified one-step hydrothermal route and CMC is firstly used as the binder for the ZnS based anode. With an average diameter of 13.4 nm, ZnS nanoparticles are uniformly anchored on rGO nanosheets. l-cysteine plays an indispensible role during the whole fabrication process as it not only serves as the sulfur source for ZnS but also acts as the reducing agent for rGO. The incorporation of rGO and ZnS has successfully alleviated the severe aggregation of ZnS nanoparticles. Besides, rGO has also endowed the composites with higher electronic conductivity and structural flexibility. The CMC binder can provide a strong binding effect to further stabilize the electrode, thus guaranteeing a higher capacity retention. Owing to these beneficial features, the CMC binded ZnS–rGO nanocomposites can retain a high discharge capacity of 705 mA h g^−1^ at 500 mA g^−1^ for 150 cycles. The excellent electrochemical performance fully demonstrates that the ZnS–rGO nanocomposites are quite promising as high-performance anode materials for next generation Li-ion batteries. And the idea of adding l-cysteine as the sulfur source and reducing agent to simplify the synthesis procedure can be extended to fabricate other metal sulfides–rGO composites.

## Conflicts of interest

There are no conflicts to declare.

## Supplementary Material

RA-008-C8RA00470F-s001
