# Supplementary material for: One-step in situ growth of ZnS nanoparticles on reduced graphene oxides and their improved lithium storage performance using sodium carboxymethyl cellulose binder
Source: RSC Adv. 2018 Mar 1;8(17):9125–33. doi: 10.1039/c8ra00470f (PMC9078578; doi:10.1039/c8ra00470f)
Supplement: RA-008-C8RA00470F-s001 [file RA-008-C8RA00470F-s001.pdf]

Electronic Supplementary Material (ESI) for RSC Advances.
This journal is © The Royal Society of Chemistry 2018

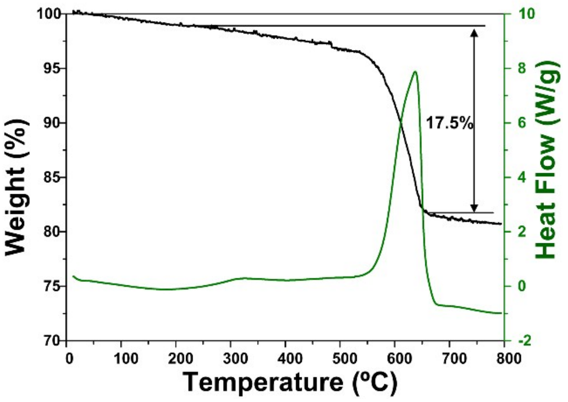

Fig. S1 TGA and DSC curves of ZS.

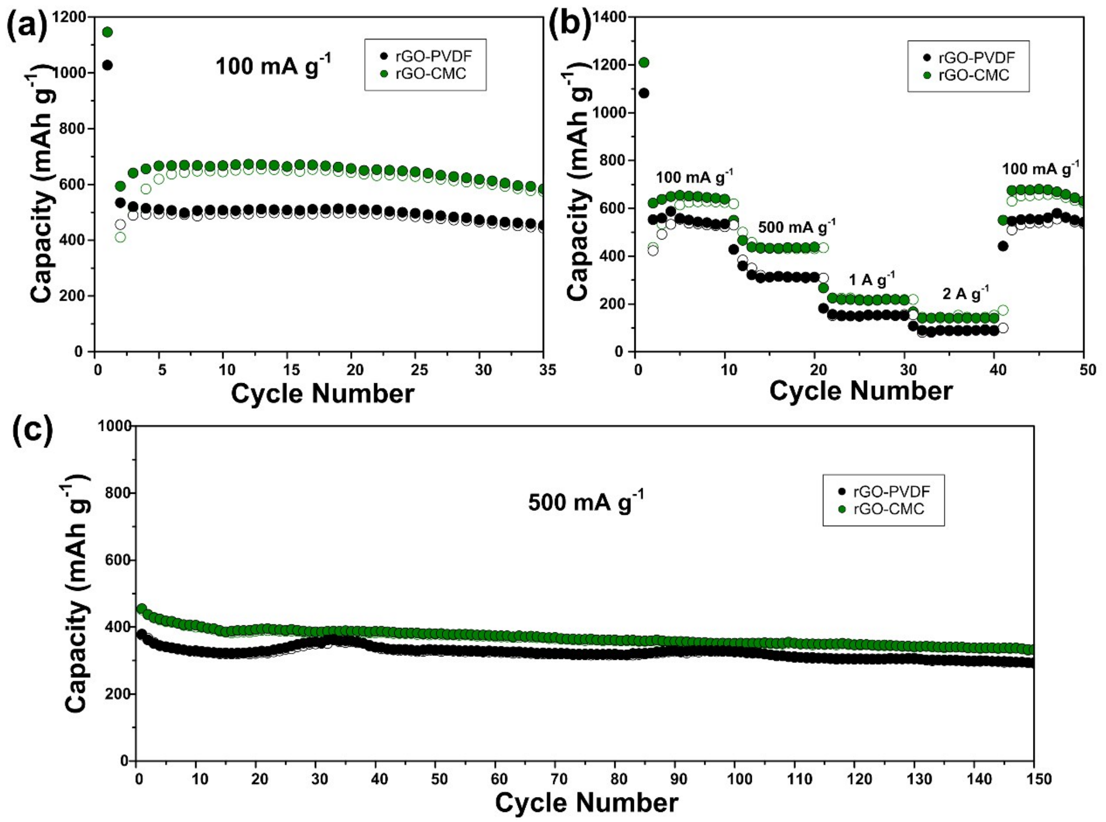

Fig. S2 (a) Cycling performance and (b) rate capability of rGO-PVDF and rGO-CMC. (c) Continuous long-term cycling performance of rGO-PVDF and rGO-CMC after the rate test.
